# Proof-of-concept study of compartmentalized lung ventilation using system for asymmetric flow regulation (SAFR)

**DOI:** 10.3389/fmedt.2023.1121674

**Published:** 2023-03-30

**Authors:** Igor Barjaktarevic, Glen Meyerowitz, Onike Williams, I. Obi Emeruwa, Nir Hoftman

**Affiliations:** ^1^ Division of Pulmonary and Critical Care Medicine, University of California Los Angeles, CA, United States; ^2^ UCLA Biodesign, University of California Los Angeles, Los Angeles, California, CA, United States; ^3^ Department of Anesthesiology, University of California Los Angeles, CA, United States

**Keywords:** compartmentalized lung ventilation, asymmetric lung injury, lung heterogeneity, acute respiratory distress syndrome (ARDS), system for asymmetric flow regulation

## Abstract

Asymmetrical distribution of acute lung injury in mechanically ventilated patients can result in a heterogeneity of gas distribution between different regions, potentially worsening ventilation-perfusion matching. Furthermore, overdistension of healthier, more compliant lung regions can lead to barotrauma and limit the effect of increased PEEP on lung recruitment. We propose a System for Asymmetric Flow Regulation (SAFR) which, combined with a novel double lumen endobronchial tube (DLT) may offer individualized lung ventilation to the left and right lungs, better matching each lung's mechanics and pathophysiology. In this preclinical experimental model, the performance of SAFR on gas distribution in a two-lung simulation system was tested. Our results indicate that SAFR may be a technically feasible and potentially clinically useful although further research is warranted.

## Introduction

The left and right lungs consist of approximately 480 million alveoli divided between them (1). In normal conditions, spontaneous breathing, characterized by negative pressure ventilation, allows for optimal gas distribution which is matched by perfusion. In settings of pulmonary airway, parenchymal or vascular pathology leading to significant oxygenation and/or ventilation compromise and acute respiratory failure, invasive mechanical ventilation is used as one of the ultimate therapeutic approaches and a bridge to recovery (2). Mechanical ventilation is based on a gas delivery using positive pressure throughout the respiratory cycle, thus allowing for a lung recruitment and optimization of ventilation and perfusion matching. Standard methodology of invasive mechanical ventilation treats the lungs as a single organ rather than targeting regional differences with individualized treatments most suitable for their unique pathophysiology. Atelectasis, infiltrates, lung masses, pleural effusions, emphysema, fibrotic changes or other musculoskeletal problems demonstrate asymmetric distribution of pathologic changes between the lung areas and which may lead to heterogeneity of ventilation (3, 4). Consequently, the gas delivered by the ventilator is distributed asymmetrically between the lungs based on their individual compliance and resistance (5). In order to maximize oxygenation and/or ventilation in mechanically ventilated patients with acute lung injury, increasing positive end-expiratory and/or driving pressures are utilized to recruit lung zones with poor ventilation/perfusion ratios (6, 7). However, these efforts often result in hyperinflation of more compliant lung zones with minimal effect on low-compliance areas, consequently resulting in additional lung injury, worsened ventilation heterogeneity and overall lack of significant improvement of V/Q ratio (8).

In order to improve lung function and deliver compartmentalized, precise regional ventilation better suited for asymmetric lung injury, we have developed a System for Asymmetric Flow Regulation (SAFR) (9). Here we report the results of a series of experiments evaluating the efficacy of this system in a lung ventilation simulation model. We hypothesize that SAFR, used together with a novel double lumen endobronchial tube (DLT) (www.uspto.report/patent/app/20200188621) can deliver compartmentalized lung ventilation with personalized and precise tidal volume delivery to each lung.

## Methods

A set of experiments was conducted at the David Geffen UCLA School of Medicine Simulation Center. Our bench set up included: (1) Puritan-Bennet model 980 ventilator (Medtronic), (2) SAFR system prototype with intraluminal balloons, (3) two parallel endotracheal tubes (ETT), each attached to a separate limb of SAFR, (4) two independent high-fidelity lung simulators (ASL 5000 Breathing Simulators, IngMar Medical). Most experiments utilized volume control assisted mode with standard and consistent parameters including tidal volume size, respiratory rate, inspiratory flow rate (decelerating), and PEEP. Ventilator output metrics, including Peak airway pressures (Ppeak) were monitored during the entire experiment.

**Figure 1:**
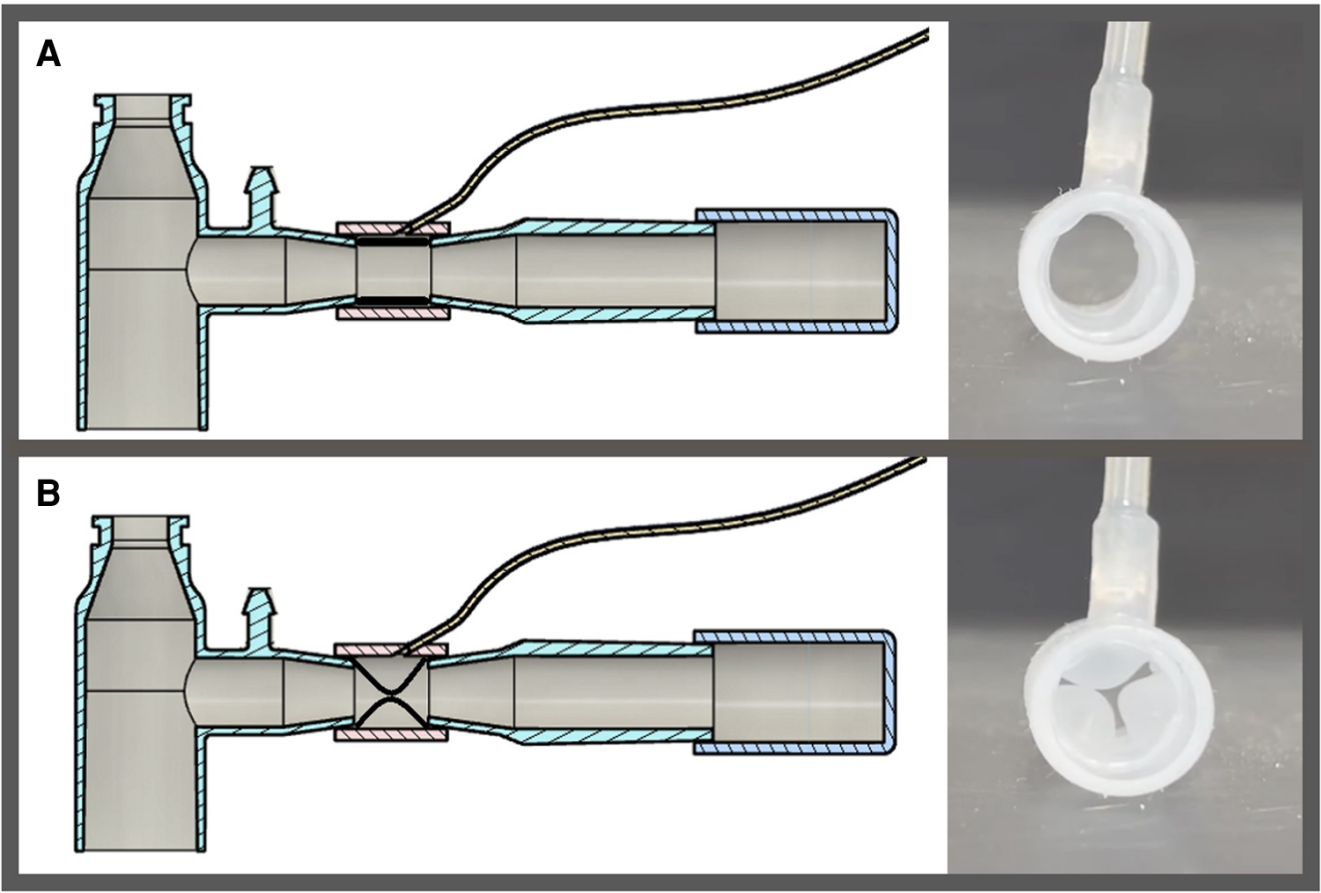
Schematic presentation of system for asymmetric flow regulation (SAFR). Each of the two SAFR's limbs contains an internal balloon whose degree of inflation regulates local gas flow and thus tidal volume delivered to each individual lung. The balloons are: (**A**) deflated at the baseline state, and (**B**) inflated during the inspiratory gas flow.

**Figure 2:**
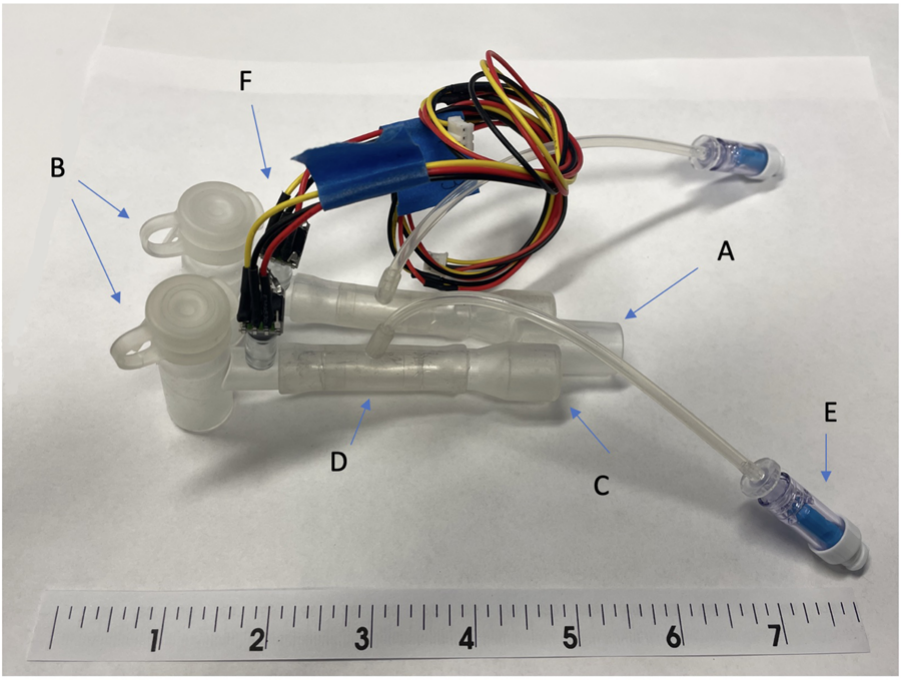
The early prototype of system for asymmetric flow regulation (SAFR). (**A**) Proximal end terminates at the connection with the mechanical ventilator circuit (**B**) Body of the SAFR branches into two channels, each containing the Flow Regulating Inner Balloon (**C**) Two distal ends connect to the double lumen tubes’ proximal (outside) outlets (**D**) Flow Regulator Inner Balloon control valve inside each of the two SAFR limbs. A Flow has capacity to occlude both lumens completely allowing for static pressures assessment and change the lumen effective diameter and surface area in each of the lumens which allows for control over uneven distribution of gas flow coming from the ventilator. (**E**) Flow Regulator inflation valve connects to the Flow Regulation external pneumatic system which controls precise Inner Balloon inflation and deflation during the respiratory cycle. (**F**) Pressure sensors and/or end-tidal CO_2_ micro-sensors are embedded into the walls of each lumen distally and connected to the outer Monitor *via* wires embedded in the tube connecting distal sensors to proximally externalized Monito.

The SAFR device is designed to be placed between the ventilator circuit (universal 15 mm female connector) and the two proximal limbs of the DLT (15 mm universal male connectors). Each of SAFR's limbs contains an internal balloon whose degree of inflation regulates local gas flow and thus tidal volume delivered to each individual lung. The system's design enables cycling (inflation/deflation) of the balloons during the inspiratory cycle while providing real-time feedback on airway pressures (dynamic/static) and overall quality of ventilation in each lung (Figures 1, 2).

For the purpose of this set of experiments, we used a simplified version (Figure 2), testing its capacity to precisely distribute volumes. We used two regular single-lumen size 7.0 endotracheal tubes (ICU Medical, San Clemente, CA) to directly deliver gas to the simulated lungs. Lung simulation system (ASL 5000 Breathing Simulators, IngMar Medical, Pittsburgh, PA) allowed for real-time modeling of airway pressures and lung dynamics in a two-lung asymmetric injury model, providing a wide range of compliance and resistance values. We used compliance ranges from 25 to 50 milliliters per centimeters of water (ml/cm H_2_O) and resistance ranges from 5 to 50 centimeters of water per liter per second (cm H_2_O/L/s). Calibration of the lung simulators was performed by the manufacturer per their recommendations. An isolation valve was located upstream of each breathing simulator to provide greater control of air flow. These valves allowed for the creation of independent airway pressure for each lung, thus allowing for the measurement of a plateau pressure at the end of the inspiratory phase. A tee fitting was used to join the two breathing simulators to a standard ventilatory breathing circuit and ventilator.

All data were collected from several different channels utilizing internal device sensors at 512 Hz and analyzed using Python scripts (ASL 5000 Software version 3.6). Flow, pressure, and volume data, as well as static compliance and resistance, were collected from the breathing simulators. Analysis scripts that align the pressure sensor readings between the two breathing simulators were used to ensure proper time synchronization.

## Results

### Baseline settings before activation of SAFR

The mechanical ventilator was adjusted with the following settings: (1) Assist control-volume control mode, (2) tidal volume 500 ml, (3) PEEP 5 cmH_2_O, (4) inspiratory gas flow 50l/min, (5) respiratory rate 18/min. The lung model was set to the following parameters: (1) lung compliance (50 ml/cm H_2_O), (2) airway resistance (5 cm H_2_O/L/s). The simulation model was registering equal distribution of tidal volume (238.3 ± 3.1 ml) in each lung. Addition of the SAFR module into the circuit did not alter the delivered tidal volumes, nor did it change the percentage circuit leak (0%–1%). The measured variation from breath to breath of 0.2% reflected a minimal and normally expected variability.

### SAFR-guided redistribution of the volumes

**Figure 3:**
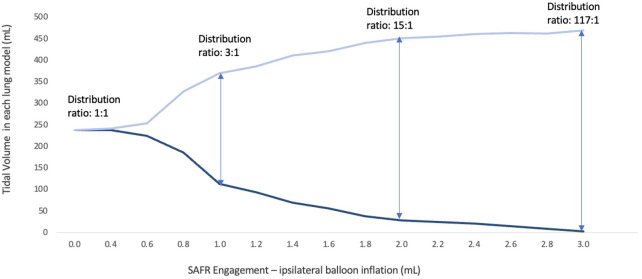
Volume distribution between the two lung models with a gradual SAFR engagement using tapered intraluminal balloon inflation.

As presented in Table 1, SAFR's intraluminal balloons were inflated in an escalating fashion in 0.2 ml increments (range 0.0–3.0 ml). This inflation in turn directly correlated with a graduated redistribution of tidal volumes between the two lungs, from 50%–50% to 0%–100% (Spearman's rho = −0.999, *p* < 0.0001). At 0.4 ml intraluminal balloon inflation, only 1.7% of tidal volume was diverted to the contralateral lung, whereas at maximal inflation (3.0 ml) 98.3% of tidal volume was diverted contralaterally. At an intermediate 1.0 ml inflation setting, the SAFR reduced tidal volume to the ipsilateral lung by 52.3% of its original volume, distributing the volumes between the lungs in approximately a 25%–75% split (113.7 ± 26.4 vs. 370.3 ± 24.7 ml for ipsilateral and contralateral lung, respectively) (Figure 3).

**Table 1:** SAFR-guided redistribution of the volumes.

TEST ID	Compliance each lung (ml/cm H_2_O)	Resistance each lung (cm H_2_O/L/s)	Balloon inflation (ml)	Mean volume Ipsilateral lung, mean (SD)	Mean Volume Contralateral lung, mean (SD)	Mean Ppeak (cm H_2_O)
1	50	5	0.0	238.3 (3.2)	238.3 (3.2)	15.7 (0.6)
2	50	5	0.4	238.3 (3.1)	242.3 (5.0)	16.0 (0)
3	50	5	0.6	225.3 (8.7)	254 (6.1)	16.0 (0)
4	50	5	0.8	186.0 (5.0)	327.7 (51.5)	17.0 (0)
5	50	5	1.0	113.7 (26.4)	370.3 (26.4)	18.3 (0.6)
6	50	5	1.2	94.7 (5.7)	387 (5.7)	19.0 (0)
7	50	5	1.4	69.7 (13.5)	412.3 (13.5)	19.7 (1.2)
8	50	5	1.6	57.3 (13.3)	421.7 (13.3)	20.3 (0.6)
9	50	5	1.8	39.0 (6.6)	441.0 (6.6)	20.7 (1.2)
10	50	5	2.0	29.3 (3.8)	452.0 (3.8)	21.3 (0.6)
11	50	5	2.2	25.0 (2.6)	455.3 (2.6)	21.3 (0.6)
12	50	5	2.4	21.7 (1.5)	461.3 (1.5)	21.3 (0.6)
13	50	5	2.6	15.0 (1.0)	463.7 (1.0)	21.3 (0.6)
14	50	5	2.8	9.7 (8.7)	462.7 (1.2)	22.0 (0)
15	50	5	3.0	4 (0.0)	470.0 (6.9)	22.0 (0)

At the stated intermediate (1 ml inflation) setting, results presented in the Table 2A indicate that adjusting tidal volume between 300 and 600 ml did not impact the L/R redistribution ratio, which remained in a narrow range (2.3:1–2.4:1). Furthermore, the distribution of volumes showed good reproducibility in a set of experiments engaging the system with the same volume (1 ml) on three occasions (interclass coefficient, ICC = 0.80 in ipsilateral and ICC = 0.87 in the contralateral lung model). Increasing levels of PEEP also did not impact the tidal volume distribution between lungs, although peak inspiratory pressures were expectedly increased, Table 2B. Finally, while locking ventilator settings at tidal volume 500 ml, rate 18, and PEEP 5, increasing inspiratory flow rates did not affect tidal volume distribution but did increase peak inspiratory pressure, Table 2C. Throughout the experiment, breath to breath variability was unaffected compared to baseline (1.2% contralateral lung, 4.5% ipsilateral lung).

**Table 2:** The effect of tidal volume, flow rate and PEEP changes on the SAFR-guided gas distribution.

Table 2A	TV (ml)	PEEP (cmH_2_O)	Flow (L/min)	Rate (1/min)	Compliance each lung (ml/cm H2O)	Resistance each lung (cm H2O/L/s)	Balloon inflation (ml)	Volume Ipsilateral Lung (ml)	Volume Contralateral lung (ml)	Distribution ratio	
1	300	5	50	18	50	5	1.0	90	208	2.3:1	
2	350	5	50	18	50	5	1.0	102	242	2.4:1	
3	400	5	50	18	50	5	1.0	118	276	2.3:1	
4	450	5	50	18	50	5	1.0	129	311	2.4:1	
5	500	5	50	18	50	5	1.0	144	344	2.4:1	
6	550	5	50	18	50	5	1.0	158	378	2.4:1	
7	600	5	50	18	50	5	1.0	172	411	2.4:1	
Table 2B	TV (ml)	PEEP (cmH_2_O)	Flow (L/min)	Rate (1/min)	Compliance each lung (ml/cm H_2_O)	Resistance each lung (cm H_2_O/L/s)	Balloon inflation (ml)	Volume Ipsilateral Lung (ml)	Volume Contralateral lung (ml)	Distribution ratio	Ppeak (cm H_2_O)
1	500	5	40	18	50	5	1.0	137	346	2.5:1	15
2	500	5	50	18	50	5	1.0	137	346	2.5:1	18
3	500	5	60	18	50	5	1.0	137	346	2.5:1	22
4	500	5	70	18	50	5	1.0	137	346	2.5:1	25
Table 2C	TV (mL)	PEEP (cmH_2_O)	Flow (L/min)	Rate (1/min)	Compliance each lung (ml/cm H_2_O)	Resistance each lung (cm H_2_O/L/s)	Balloon inflation (ml)	Volume Ipsilateral Lung (ml)	Volume Contralateral lung (ml)	Distribution ratio	Ppeak (cm H_2_O)
1	500	5	50	18	50	5	1.0	137	346	2.5:1	18
2	500	7.5	50	18	50	5	1.0	137	346	2.5:1	20
3	500	10	50	18	50	5	1.0	137	346	2.5:1	22
4	500	12.5	50	18	50	5	1.0	137	346	2.5:1	24
5	500	15	50	18	50	5	1.0	137	346	2.5:1	27

### Impact of balloon engagement on dynamic ventilatory pressures

SAFR engagement with balloon inflation increased dynamic resistance and consequently led to the increase of dynamic pressures sensed by the main ventilator. Peak airway pressures gradually increased with escalating balloon inflation, and this positive correlation was highly significant (Spearman's rho = 0.99, *p* < 0.001). Peak pressure values at maximal SAFR settings increased by 40% from the pre-inflation baseline value of 15.7 ± 0.6 cmH_2_O, although the absolute value of 22.0 cmH_2_O was still within acceptable limits. As previously mentioned, increasing PEEP and inspiratory gas flows also increased peak pressures measured at the ventilator.

### The effect of volume correction in asymmetric lung injury

To test SAFR’s performance in an asymmetric lung model, we simulated two distinct scenarios: (1) differential airway resistance between left and right lung, and (2) differential compliance between left and right lung.

In the first experiment, we set the two simulators to different airflow resistances (50 vs. 5 cm H_2_O/L/s), while leaving simulator compliance unchanged (50 ml/cm H_2_O each). Prior to SAFR activation tidal volume was preferentially delivered to the low resistance lung (73.3% tidal volume) compared to the to the high resistance side (26.7% tidal volume). Activation of SAFR with balloon inflation (0.8 ml) to the low resistance lung rebalanced tidal volume distribution to a roughly even distribution (48.8% vs. 51.2%), whereas balloon inflation to 3 ml led to nearly complete tidal volume delivery (94.1%) to the high-resistance lung (Table 3A).

**Table 3:** The effect of volume correction in asymmetric lung injury.

Table 3A	Compliance each lung (ml/cm H_2_O)	Resistance in the left lung (cm H_2_O/L/s)	Resistance in the right lung (cm H_2_O/L/s)	Balloon inflation (ml)	Volume Ipsilateral Lung (ml)	Volume Contralateral lung (ml)	Distribution ratio
1	50	50	5	0	120	333.3	1:2.8
2	50	50	5	0.8	218.3	228.0	1:1.1
3	50	50	5	1	238.5	206	1.2:1
4	50	50	5	2	363.6667	70.33333	5.2:1
5	50	50	5	3	405	25	16.2:1
Table 3B	Compliance Left lung model (ml/cm H_2_O)	Compliance Right lung model (ml/cm H_2_O)	Resistance in both lung models (cm H_2_O/L/s)	Balloon inflation (ml)	Volume Ipsilateral Lung (ml)	Volume Contralateral lung (ml)	Distribution ratio
1	25	50	5	0	175	280.7	1:1.6
2	25	50	5	0.6	189.3	268.3	1:1.4
3	25	50	5	0.7	217	243.5	1:1.1
4	25	50	5	0.8	268.3	188.7	1.4:1
5	25	50	5	1	285.3	167.3	1.7:1
6	25	50	5	2	407.3	38	10.7:1

In the second experiment, we set the two simulators to different lung compliance (25 vs. 50 ml/cm H_2_O), while leaving simulator airway resistance unchanged (5 cm H_2_O/L/s in both lung models). Prior to SAFR activation tidal volume was preferentially delivered to the normal compliance lung (61.6% tidal volume) compared to the low compliance lung (38.4% of total volume). Activation of SAFR with balloon inflation (0.7 ml) to the normal compliance lung rebalanced tidal volume distribution to a roughly even distribution (47.2% vs. 52.8%), whereas balloon inflation to 2.0 ml led to nearly complete tidal volume delivery (91.2%) to the low compliance lung (Table 3b).

## Discussion

In the series of experiments reported in this analysis, we evaluated a novel system for asymmetric flow regulation of mechanical ventilation. We confirm that delivery of precise and customized tidal volume to each lung can be achieved using a single standard mechanical ventilator when combined with the SAFR device and a double lumen endobronchial tube.

Our report addresses several relevant and timely issues related to mechanical ventilation. First, current mechanical ventilation strategy treats the lungs as a single unit, although clinically pathophysiologic heterogeneity is both common and well recognized. For example, ARDS with persistent hypoxemia is often treated with alternating patient positioning between prone and supine to improve airflow distribution and V/Q matching (10). Unfortunately, the ability to address the same problem independent of the gravity effect has been minimal. Although asymmetrical lung injury is not infrequent, attempts to recruit atelectatic lung zones with PEEP treat the entire lung unit uniformly. Such a strategy may cause overdistension and barotrauma in healthy, compliant lung zones, without improving V/Q matching in the injured, low-compliant zones (8). A further consequence is the diversion of pulmonary blood flow away from healthy lung zones to diseased ones, thereby worsening shunt, hypoxemia and lung injury (11). A review of isolated lung ventilation (ILV) by Berg et al. suggests that the institution of individualized lung ventilation (ILV) may reduce volutrauma and shunting in the healthier lung while promoting alveolar recruitment in the diseased lung (12). The authors further discuss the benefits of ILV in clinical scenarios with asymmetrical or unilateral pathopyshiology including: (1) pneumonia, (2) persistent bronchopleural fistula, (3) pulmonary hemorrhage, (4) pulmonary contusion, (5) primary graft dysfunction following single lung transplantation. Use of ILV could possibly decrease the need for more costly, invasive, and morbid strategies such as ECMO, especially in scenarios where such pathways are unavailable (limited expertise or resources) or contraindicated (profound thrombocytopenia, disseminated intravascular coagulation, recent tPA use).

Lung ventilation heterogeneity has been gaining recognition given recently discovered associations with clinical outcomes in both acute and chronic respiratory conditions (9, 13, 14). The SAFR method focuses on management of patients with hypoxemic and/or hypercapnic respiratory failure with concomitant asymmetrical distribution of injury between the two lungs. Successful integration of SAFR into the mechanical ventilation circuit requires safe and effective split lung ventilation. A prerequisite for achieving lung isolation is the double lumen endobronchial tube, a specialized airway device used for over 60 years in clinical medicine, though mostly in thoracic surgery (12). These tubes, while enabling differential lung ventilation, have several disadvantages when compared to standard endotracheal tubes. First, they are large and rigid which increases the risk and difficulty of endotracheal intubation, and thus require special training and expertise. Second, their internal lumens are small, making pulmonary toilet all but impossible. Third, these devices require exact positioning but are easily dislodged, requiring closer monitoring and shorter-term use (15). Clinically, DLTs are usually used in critical care settings only for temporary single-lung ventilation (i.e., after lung surgery), and have not been traditionally used for fine-tuning of the ventilation distribution in medical patients with respiratory failure. Nevertheless, the development of a novel double lumen tubes with specialized features may allow for wider clinical use beyond thoracic surgery, offering an opportunity to develop compartmentalized lung ventilation modalities. Such a DLT will enable easy and safe intubation given its soft materials and small external size. Its universal design will enable exact fit into either mainstem bronchus ensuring stability and reducing dislodgement. Furthermore, its patented design will allow therapeutic bronchoscopy through both lumens, ensuring the needs of chronically vented patients.

This study reports early pre-clinical results of a novel system exploring the concept of compartmentalized lung ventilation. SAFR represents a feasible theoretical approach to lung ventilation which allows physicians to substantially redistribute gas flow between the lungs based on their understanding of lung pathophysiology in mechanically ventilated patients. Resulting change in tidal volumes could protect an injured or vulnerable lung (i.e., hemorrhage, post-single lung transplant) while also recruiting a poorly compliant lung (i.e., unilateral pleural effusions, bronchopleural fistula). Precise gas flow regulation in each limb, based on the carefully titrated inflation of internal balloons, leads to delivery of unique tidal volumes to each lung. This unique redistribution of ventilation between the two lungs is not altered by changing PEEP, tidal volumes, or inspiratory flow rates. Future iterations of SAFR with continuous monitoring (i.e., ventilation pressures and end-tidal CO_2_) and feedback-driven automation could enable treating physicians to guide ventilation management based on the dynamic nature of critical illness.

The reported findings are limited by the preclinical, proof-of-concept nature of this study design. Potential to compartmentalize ventilation between the two lungs using SAFR does not translate into the ability to correct ventilation heterogeneity that may be inherent to either of the lungs. SAFR is a novel device concept currently in an early stage of development. Given that the experiment was conducted on in-vitro lung simulators, actual effects on real lung tissue cannot be inferred. Furthermore, its dynamic impact on the cardio-respiratory interaction with concomitant hemodynamic effects were not simulated and therefore not accounted for.

## Conclusion

Compartmentalized lung ventilation using a regular mechanical ventilator and a double lumen endobronchial tube is theoretically feasible if a system for asymmetric flow regulation (SAFR) is applied. This system allows for a precise and substantial volume redistribution to each lung, allowing an opportunity to better manage lung ventilation heterogeneity. More studies are necessary to improve our understanding of the clinical potential of this novel approach.

## Data Availability

The raw data supporting the conclusions of this article will be made available by the authors, without undue reservation.
